# The state of play of rodent models for the study of Clostridioides difficile infection

**DOI:** 10.1099/jmm.0.001857

**Published:** 2024-07-19

**Authors:** Anaïs Brosse, Héloïse Coullon, Claire Janoir, Séverine Péchiné

**Affiliations:** 1 Micalis Institute, Université Paris-Saclay, INRAE, AgroParisTech, Jouy-en-Josas, France

**Keywords:** animal models, *Clostridioides difficile*, conventionally reared mice, gnotobiotic mice, hamsters, relapse model

## Abstract

*Clostridioides difficile* is the most common cause of nosocomial antibiotic-associated diarrhoea and is responsible for a spectrum of diseases characterized by high levels of recurrence and morbidity. In some cases, complications can lead to death. Currently, several types of animal models have been developed to study various aspects of *C. difficile* infection (CDI), such as colonization, virulence, transmission and recurrence. These models have also been used to test the role of environmental conditions, such as diet, age and microbiome that modulate infection outcome, and to evaluate several therapeutic strategies. Different rodent models have been used successfully, such as the hamster model and the gnotobiotic and conventional mouse models. These models can be applied to study either the initial CDI infectious process or recurrences. The applications of existing rodent models and their advantages and disadvantages are discussed here.

## Introduction

*Clostridioides difficile* is a Gram-positive, anaerobic, spore-forming and toxin-producing bacterium responsible for a wide range of diseases from antibiotic-associated diarrhoea to pseudomembranous colitis. Recurrence occurs in 25 % of patients after a primary infection, and a patient with a recurrence is at a much higher risk of subsequent episodes. Infection with * C. difficile* is acquired through the transmission of spores that are ingested. Once in the gut, bile acids (BAs) play an important role in inducing the germination of spores into metabolically active vegetative cells. The main protective barrier against *C. difficile* infection (CDI) is the normal gut microbiota. In the case of dysbiosis, which is induced mainly by antibiotics, this opportunistic pathogen colonizes the colon. The pathology of CDI results from the expression of a number of *C. difficile* virulence factors, including toxins, toxin A (TcdA) and toxin B (TcdB), and for some strains, binary toxin (CDT) and surface proteins. The toxins disrupt tight junctions and destroy the actin cytoskeleton of enterocytes, resulting in the breakdown of intestinal barrier integrity and loss of absorptive function. The toxins induce an inflammatory response by recruiting neutrophils and mastocytes, which release cytokines. The inflammatory process finally leads to the formation of pseudomembranes in the colon.

Developing experimental animal models to study the entire *C. difficile* infection process in a way that best mimics what happens in humans is a real challenge. A wide range of animal models of *C. difficile* have been developed, and these have been essential in providing insight into disease pathophysiology, including colonization and environmental factors such as diet or microbiome and potential treatment options.

Mammalian models are commonly used as the gold standard to study the pathogenesis of CDI. However, alternative models are needed due to the cost and ethical considerations related to mammalian animal models. In addition, such models are not suited for higher throughput experiments. Several alternative models are currently emerging: (i) nematodes (*Caenorhabditis elegans*), recommended for high-throughput screening of anti-*C. difficile* compounds; (ii) fruit fly (*Drosophila melanogaster*), useful to study the mechanisms of CDI-associated host immune response and regulation; (iii) moth larva (*Galleria mellonella*), used to study pathogenicity and virulence of *C. difficile* and (iv) zebrafish (*Danio rerio*), which allow the visualization of the interplay between *C. difficile* and host cells as well as the *in vivo* toxic effect of toxins.

In this review, we will focus on rodent models of CDI. Until recently, the ‘gold standard’ model for CDI was considered to be the Syrian golden hamster. However, mouse models, either conventional mice or gnotobiotic mice, are also widely used to study the different stages of pathogenesis, from spore germination, host colonization and toxin production and histopathology, inflammatory processes and interaction with microbiota. Gnotobiotic mice are axenic (germ-free) animals, which are inoculated with defined bacterial strains, alone or with a cocktail of strains. Pre-exposure to antibiotics is dispensable in gnotobiotic mice, depending on the strain used to induce infection. In contrast, and similarly to humans, both conventionally reared mice (conventional mice) and hamsters require pre-treatment with antibiotics to modify the microbiota such that subsequent exposure to *C. difficile* results in clinical manifestations and significant tissue lesions [1,2]. As recurrent CDI is a major clinical problem and very challenging to treat, it is useful to perform experiments in animal models of relapse/recurrence. The aim of this review is to give an update on the different rodent models and their use for different purposes, their advantages and disadvantages.

## Hamster models

### Clinical signs and histopathology

The Syrian golden hamster is often regarded as a relevant small animal model of *C. difficile* disease as oral infection of animals pre-treated with antibiotics generates many of the symptoms observed in humans. These include colonization of the large bowel, diarrhoea (wet tail and loose faeces), histological pathologies and sporulation of the organism [3]. In addition to the general deterioration of the animal, there are changes in the appearance of the gastrointestinal tract, particularly in the caecum. Redness, inflammation, fluid accumulation and enlargement are usually seen, accompanied by a decrease in gut motility [4]. However, the site of *C. difficile* infection in hamsters is different to that in humans, occurring mainly in the caecum rather than the colon. In addition, the mortality rate in hamsters is close to 100 %, which is very different from that in humans. Nevertheless, hamsters remain a clinically relevant model for studying the *C. difficile* pathogenesis and testing new vaccines and therapeutics [2]. If left untreated, fulminant disease ensues, and death is usually within 48 h of inoculation [5]. Of note, country-specific animal welfare legislation can require investigators to intervene before hamsters can succumb to the natural course of the disease, which proves difficult given the speed at which CDI occurs in hamsters [4]. Researchers should, therefore, ensure that they respect the animal welfare legislation applicable to their project. More recently, attempts have been made to refine the model to extract maximal data while minimizing animal suffering. This was achieved by using a combination of qualitative and quantitative measurements taken during infection and postmortem. More appropriate and frequent monitoring of animals during the acute phase of infection is now conducted, with more appropriate endpoints for these experiments, such as significant loss of weight or decreased activity [3]. Sex does not appear to be an important factor in all studies conducted in the hamster model, although more and more studies are being conducted using mixed-sex hamsters [6]. Most hamsters weigh between 80 and 100 g at the start of the experiments.

### Induction of dysbiosis

CDI requires prior induction of dysbiosis, often through antibiotic therapy. In 1968, Small initially demonstrated that lincomycin produced enterocolitis when given to hamsters [7]. A few years later, Larson and Borriello (1990) compared several antibiotics for their propensity to induce CDI in hamsters. Infection occurred after administration of very low doses of ampicillin, clindamycin, flucloxacillin or cefuroxime, with little difference in the degree of susceptibility they induced. However, of all the antibiotics tested, clindamycin induced a longer period of susceptibility to infection [8]. Later, Merrigan *et al*. investigated the effect of ampicillin and ceftriaxone compared to clindamycin on the susceptibility of hamsters to infection after challenge with *C. difficile*, specifically strain B1. The antibiotics tested differed in the duration of CDI susceptibility induced. Hamsters were found to remain susceptible to CDI for at least 4 days after ampicillin and clindamycin, but for a shorter period after treatment with ceftriaxone [9]. Furthermore, clindamycin was very effective in accelerating colonization and disease progression. For this reason, most studies in the hamster model use clindamycin. Doses vary from 10 to 50 mg kg^−1^, and the interval between antibiotic exposure and challenge varies from 7 days pre-challenge to 1 day post-challenge [10,11]. It is important not to delay challenge beyond a week after antibiotic treatment, as the microbiota is quickly restored. It was found that animals infected with strain B1 on day 7 after clindamycin became colonized and succumbed to CDI, but animals infected on days 14, 21 or 28 after antibiotic administration were not colonized and remained healthy [9].

The most commonly used protocol today for inducing CDI in the hamster model is pre-treatment with oral clindamycin (30 mg kg^−1^) 5 days before *C. difficile* challenge. However, other antibiotics have been used, such as moxifloxacin [12], cefoxitin [13] or ampicillin [14]. Antibiotics can be administered by several routes, orally as well as parenterally (subcutaneously, intramuscularly or intraperitoneally), which does not seem to impact gut dysbiosis [10,15, 16]. To enhance challenge, animals can be further given sodium bicarbonate to reduce stomach acidity by approximately 10 min prior to challenge [17]. Usually, antibiotic treatment is based on a single dose of clindamycin; however, some studies have used 3 consecutive days of administration just before challenge [18]. To characterize antibiotic-induced dysbiosis, Miezeiewski *et al*. performed longitudinal molecular profiling on the hamster microbiome and found specific genera of gut bacteria that were suppressed in growth after a single dose of clindamycin at an early time point. In particular, they observed marked changes across *Bacteroidetes* and *Proteobacteria*. An incomplete return to the baseline microbiome occurred by day 15, correlating with inhibition of *C. difficile* growth *in vitro* and *in vivo* [19].

### Challenge with *C. difficile*

The challenge of antibiotic-treated hamsters with *C. difficile* can be performed with spores (50 to 10^6^) or with vegetative cells (10^3^ to 10^8^). Whichever form is chosen, the outcome is acute and fatal. The dynamics of spore germination in the gastrointestinal tract of hamsters was studied by Wilson *et al.* after intragastric inoculation with spores and a 51Cr tracer. Seventy-eight per cent of the spores germinated in the small intestine within 1 h [20]. In other studies where only vegetative cells were administered, not all cells survived the harsh conditions (acidity and proteases) of the stomach [21,22]. Since the natural course of infection is by the ingestion of *C. difficile* spores, more recent studies deploy spore inoculations [23,24].

### Applications

Early infection studies aimed to investigate the spectrum of human disease caused by *C. difficile* and showed that strains could be classified into highly virulent or less virulent categories based on how quickly the infected hamsters succumbed to disease [25]. The hamster model was then used to compare the virulence of different animal and human isolates. Over time, the hamster model has been used to study all the stages of *C. difficile* pathogenesis: germination of spores, colonization and the role of toxins or toxin components. For example, it was unknown whether it is necessary for the spore to attach to the gut before germination. Hong *et al.* investigated how the spore coat protein CotE could facilitate host colonization by *C. difficile* [26]. They showed that when CotE is absent, both colonization and virulence were markedly reduced. They demonstrate that the attachment of spores to the intestine is essential for the development of CDI. Trzilova *et al.* analysed the effect of flagellum and toxin phase variation on intestinal colonization and disease development [27]. Their intriguing results showed that the ability of *C. difficile* to phase vary flagella and toxins influences colonization and disease development and suggest that flagella phenotypic variants have altered capacities to cause disease.

Simpson *et al.* showed that the binary toxin-binding component, CDTb of *C. difficile,* contributes significantly to disease in the hamster model. Furthermore, they found that complementation of a CDT-deficient *C. difficile* strain with CDTb alone restored virulence [28].

Bilverstone *et al.* demonstrated that the glucosyltransferase (GT) activity of *C. difficile* toxin B is required for disease pathogenesis. To establish the importance of this GT activity in the pathogenesis of CDI, they generated a mutant strain of *C. difficile* that produced a GT-defective toxin. Hamsters infected with the wild-type or a mutant of TcdA, Δ*tcd*A, developed fulminant infection within 4 days; however, all hamsters infected with the GT-defective mutant survived to the experimental endpoint, the full 10 days after infection without developing any symptoms of CDI or evidence of caecal inflammation. Their findings have important implications for understanding the pathogenesis of *C. difficile* disease [23].

The hamster model is also useful for testing vaccine candidates and investigating their protective efficacy against *C. difficile* disease. A number of vaccination studies have been carried out in the hamster model, including oral immunization with non-toxigenic * C. difficile* strains expressing chimeric fragments of TcdA and TcdB [16] and oral vaccination with the colonization factor CD0873 [24] or with spore proteins [13]. In addition, the hamster model is frequently used to assess the efficacy of new therapeutic agents such as the small-molecule compound, CDBN-YGXZ [29,30]. The mechanism of action of CDBN-YGXZ was validated using a pyruvate : ferredoxin/flavodoxin oxidoreductase inhibition assay. The efficacy of CDBN-YGXZ was evaluated *in vitro* by MIC tests and efficacy in treating CDI in *in vivo*. None of the untreated animals (control group) survived 7 days post-infection; however, in the CDBN-YGXZ group, only one animal died, and during the acute phase of infection, the remaining nine animals survived and gained weight. Importantly, this new molecule appears to have minimal impact on the gut microbiota compared to classical therapeutic approaches [29].

The hamster model has also been used to explore the relationship between dietary macronutrients and antibiotic-associated CDI. Bhute *et al.* showed that most hamsters fed a high-carbohydrate diet developed fulminant CDI, and there were also several with late-onset CDI that were not observed in hamsters fed a standard laboratory diet. They speculate that prolonged high-carbohydrate-induced dysbiosis allowed *C. difficile* to persist in the gut, where it was able to multiply even after vancomycin treatment, leading to the delayed onset of infection [31].

### Limitations

Hamsters have been used since the 1970s to study pathogenesis, vaccines and treatment; however, such valuable *in vivo* studies are greatly hampered by the lack of hamster-specific reagents, notably antibodies against secretory IgA, the first line of defence against such enteric pathogens. Additionally, the lack of genetically modified hamsters makes it challenging to address research questions investigating specific host responses. Furthermore, the rapid onset of CDI in hamsters limits the window of time for exploring bacterial-host interactions. To overcome these limitations, mouse models of infection have been developed.

## Mouse models

### Gnotobiotic mouse models

#### Mono- and dixenic models: virulence, omics and competitive assays

Germ-free mice have no micro-organisms living in or on them, allowing to specifically control an animal’s microbiota through the direct inoculation of bacteria of interest. In the field of gnotobiology [from the Greek ‘gnōtos’ (known) and ‘biotic’ (life)], such animals are used to study the effects of inoculation with specific, known microbes. Gnotobiotic models offering a different degree of microbial complexity have been developed in the past, ranging from mono-colonization (monoxenic animals) or colonization with two different bacterial strains or species (dixenic animals) and even more complex communities.

The first mouse model used to study the pathophysiology of *C. difficile* was the germ-free mouse model. The first description of the monoxenic CDI model was by Onderdonk *et al*. in 1980, who showed that germ-free CD-1 mice infected with a clinical strain developed persistent colonic inflammation associated with watery stools. No other clinical signs were observed, and all mice except one survived. Treatment post-infection with vancomycin resulted in a reduction in bacterial load, demonstrating that this model can be used both as a colonization model and to test treatments [32]. Over the next decade, gnotobiotic mouse models became more widely used to study *C. difficile* virulence mechanisms but also to unravel the mechanism of the colonization resistance barrier conferred by the healthy microbiota in conventional animals. Strains of *C. difficile* were found to colonize the mice in a similar manner to that in hamsters [33]. The relationship between toxin production and morbidity and mortality was investigated in gnotobiotic mouse models and confirmed earlier observations reported in hamsters with the intensity of caecal inflammation directly attributable to toxin production. Accordingly, passive immunization of germ-free mice with monoclonal antibodies raised against TcdA was shown to protect animals from infection with the *C. difficile* VPI 10463 strain [34]. Studies in gnotobiotic mice have highlighted differences in susceptibility to infection depending on the strain used. Inoculation with low toxin-producing TcdA-/TcdB+ strains did not kill germ-free mice; furthermore, pre-infection of germ-free mice with this strain protected animals from infection following subsequent challenge with the VPI 10463 strain by reducing VPI 10463 colonization [35]. In light of these findings, gnotobiotic models have been used to successfully unravel the relationship between *C. difficile* strains and their ability to induce infection and how some strains can confer colonization resistance against others.

Around 40 years ago, studies investigating colonization resistance in germ-free mice demonstrated that pre-implantation of *Escherichia coli* or *Bifidobacterium* strains prevented clinical manifestations of CDI after subsequent challenge with *C. difficile* VPI 10463, although the bacterial load was not reduced, in contrast to the levels of faecal toxin B [36]. More recently, germ-free Swiss Webster mice pre-colonized with a murine *Lachnospiraceae* isolate had a 2-log reduction in *C. difficile* colonization and 80 % reduced mortality compared to the control groups [37]. Another recent study showed that pre-colonization of germ-free mice with the commensal *Paraclostridium bifermentans* protected animals from death and reduced intestinal *C. difficile* burden, together with toxin levels, leading to less intestinal damage after challenge with VPI 10463 compared to monoxenic control mice. This protection was related to competition for amino acids involved in the Stickland reaction (a major energy source for *C. difficile in vivo*, see below), which are consumed by *P. bifermentans* to the detriment of *C. difficile* [38].

The dixenic models are also useful to bring out synergistic relationships between *C. difficile* and specific commensal bacteria. Girinathan *et al.* showed that germ-free mice pre-colonized with a strain of *Clostridium sardiniense* develop a more severe disease post-challenge with *C. difficile* and succumb to disease more rapidly [38]. Previously, it was shown that *Bacteroides thetaiotaomicron* was able to support the growth of *C. difficile* by its ability to produce succinate as a final metabolic product of polysaccharide fermentation or to release sialic acid from mucins, both residues being nutrients that sustain the *in vivo* growth of *C. difficile* [39,40]. The study of trophic interactions and nutrient competition has thereafter mostly been conducted in conventionally reared mouse models, but the gnotobiotic model offers a simplified microbiota model for studying direct relationships between *C. difficile* and specific commensals, notably prominent members of the microbiota.

Competitive dixenic models have been particularly used to identify subtle differences in the ability of different strains to colonize the host, as measured by faecal shedding or binding levels to the caecal mucosa [41]. We used this model (C3H/He) to compare the colonization fitness of isogenic *C. difficile* mutants. As an example, we showed that the *sigB* mutant was gradually cleared from mice, unlike its parent strain 630*Δerm*. This inability to persist was attributed to reduced adhesion to caecal tissues [42]. Another example is our study comparing the colonization fitness of a mutant defective in the production of colonization factor, CD0873 and the parental strain. The unequivocal data highlight the important role of this lipoprotein in the long-term persistence of *C. difficile* in the murine intestinal tract [43]. Only severely impaired mutant strains are unable to colonize the gut of initially germ-free mice; however, germ-free mice have certain atypical characteristics, in particular a higher oxygen concentration in the caecal and colonic niches. The cysteine desulfurase IscS2 plays a role in oxygen resistance in the strictly anaerobic *C. difficile*, and the *ΔiscS2* mutant was completely defective in its ability to colonize germ-free mice [44]. To our knowledge, this is the only example of a viable *C. difficile* strain (either naturally occurring or genetically derived mutant) that is unable to colonize germ-free mice.

Monoxenic models were the first used models for genome-wide temporal transcriptomic studies. Several assays have been performed by us and others to understand the regulation of gene expression for bacterial adaptation and virulence during infection. In particular, these experiments highlight the importance of the metabolic adaptation of *C. difficile* to the host, which may differ between strains. For example, initial experiments with strains 630 or the 027 epidemic R20291, carried out in C3H/He monoxenic mice, underlined the common use of amino acids through Stickland reactions for energy production [45,46]. In both strains, transcriptomic experiments confirmed the temporal regulation of genes encoding virulence factors (e.g. *tcdA* and *tcdB*) and showed for the first time the importance of sporulation during the *C. difficile* life cycle in the host. More recently, the metabolic adaptation of *C. difficile* in the context of an inflamed or non-inflamed intestinal milieu has been analysed in detail in two very elegant studies, 3 days post-infection, in monoxenic Swiss Webster mice colonized either with the wild-type toxigenic strain (resulting in an inflamed colon) or with an isogenic non-toxigenic mutant (resulting in negligible colonic inflammation). Using other mouse models and a combination of transcriptomic and metabolomic profiling of several metabolic mutants, the authors show that *C. difficile* can manipulate its environment, using either diet or specific host-derived nutrients produced in an inflamed or non-inflamed environment [47,48]. In these studies, the authors highlight that gnotobiotic mouse models allow sufficient coverage of the *C. difficile* transcriptional profile for differential analysis of gene expression, which would be more difficult to achieve in a more complex environment.

Finally, the monoxenic model of CDI has also enabled the study of the spatial distribution of *C. difficile* in the intestinal mucosa. The association of bacteria with the epithelial surface was first documented in germ-free mice infected with the strain VPI 10463 using scanning electron microscopy [49]. More recently, studies by our group used a monoxenic mouse model to study the spatial 3D distribution of strain R20291 in intestinal tissues, suggesting that R20291 is organized in a biofilm architecture in this model [50].

#### More complex models of gnotobiotic mice: metabolic examination and therapeutic assays

The use of gnotobiotic mice has led to a greater understanding of colonization resistance by specific microbiota and further enabled the testing of therapeutics directed against *C. difficile*. Importantly, gnotobiotic mice can be pre-colonized with a defined mixture of commensal bacteria before *C. difficile* infection. The possibilities are almost endless with any species implanted depending on the scientific question to be addressed. However, in most studies to date, pre-colonization has deployed the naturally dominant commensal species of the gut microbiota [48]. There are also simplified standardized models of microbiota, such as the mouse stably associated with a simplified consortium comprising 12 murine bacterial species, named ‘oligo-mouse microbiota’ (Oligo-MM12). This model has been used to highlight the importance of the presence of secondary biliary acids, particularly deoxycholic acid (DCA), in the resistance to *C. difficile* host colonization and infection [51].

Human microbiota-associated mouse models have also been used. The first is a humanized model, resistant to CDI, obtained by colonizing germ-free animals with a healthy microbiota, either directly from humans or from faeces of previously humanized mice. Therefore, in this model, the mice must be rendered susceptible to CDI by antibiotic exposure to disrupt the colonization resistance barrier exhibited by the healthy microbiota, such as amoxicillin–clavulanic acid (3 mg per mouse) for 7 days [52]. The second model is a humanized model susceptible to CDI in which germ-free mice receive a faecal transplant with human dysbiotic microbiota. In this case, no further antibiotics are required prior to *C. difficile* infection. As mentioned earlier, this humanized model was used to confirm the importance of the availability of Stickland amino acids for the colonization process of *C. difficile* [53]. In addition, this model was also used to demonstrate the role of a plasmid encoding an amidase homologue putatively involved in cell wall integrity and vancomycin susceptibility of *C. difficile* [54].

Depending on the research question, either pooled human-derived faeces or a faecal sample from a single individual can be implanted. Human-derived gut microbial communities are given at least 2 weeks (or, more usually, 4 weeks) to equilibrate in the mouse gut after transplantation [53,55]. This is followed by the implantation of a microbiota close to that of the donor, which has been suggested to be stable over multiple generations [56]. This makes the humanized mouse model highly relevant for studies investigating interactions between *C. difficile* and the microbiota [57]. Conversely, germ-free mice transplanted with a dysbiotic faecal sample showed a gradual change in the microbiota to adapt to the new host [55].

### Other gnotobiotic models

The first germ-free animals used to study the virulence of *C. difficile* were juvenile hares and young adult rats, which develop pathological disorders associated with a mortality rate dependent on the level of toxin produced by the *C. difficile* strain [58,59]. Due to the development of gnotobiotic mouse models, which are cheaper and easier to handle, hare and rat models are no longer used. Another gnotobiotic model that has recently been developed as a preclinical model for testing new therapeutics (e.g. antibodies) is the germ-free piglet model, which has the advantage of being naturally very susceptible to CDI. However, this model is more expensive and requires approved animal facilities [60,61].

#### Advantages and disadvantages of gnotobiotic mouse models

Gnotobiotic mice, whether monoxenic, dixenic or humanized, are derived from germ-free mice. Three main mouse lineages have been used, the inbred C57BL/6 and C3H and the outbred Swiss Webster mice, but a few experiments have also been carried out with Balb/C mice. For monoxenic mice, animals are typically orally infected with vegetative cells (10^7^ to 10^8^ CFU, Colony Forming Unit) taken from exponential- or stationary-phase cultures, and the colonization level reached by *C. difficile* is around 10^8^ c.f.u. g^−1^ faeces. The outcome of infection in *C. difficile* of an initially germ-free mouse highly depends on the virulence of the strain used (ranging from moderate diarrhoea with some weight loss to rapid animal death), although toxin production is generally higher than in conventional mouse models [62]. If the mice do not die from CDI, they remain colonized throughout the experiment.

Gnotobiotic models are extremely useful because they allow the behaviour of *C. difficile* to be studied in a simplified and highly controlled environment, notably the interactions between this pathogen and one or several specific commensal species of the intestinal microbiota, without the bias of genetic diversity at least when inbred mouse lineages are used. Factors affecting host colonization by *C. difficile*, such as competition for specific nutrients, *in vivo* regulation of *C. difficile* gene expression or differences in colonization fitness between *C. difficile* strains, are some of the many results obtained, thanks to the use of these models, paving the way for a detailed understanding of *C. difficile* pathophysiology in a more complexity-controlled environment. However, gnotobiotic mouse models have two important limitations. The main practical drawback is the need for specific facilities to house these animals in a sterile environment in order to avoid any environmental contamination and to maintain a controlled microbiota. Working with these models requires meticulous protocols and good experimental controls, as well as trained personnel. Germ-free or gnotobiotic mice are bred and housed in isolators, either flexible (under mandatory positive pressure) or rigid, the latter being suitable to work under positive or negative pressure [63]. Isolators are ventilated with HEPA-filtered sterile air (High-Efficiency Particulate Air), and food, water and other supplies must be appropriately sterilized using autoclave, irradiation or treatment with disinfectants [64,65]. The germ-free status of axenic mice needs to be checked before any bacterial implantation by anaerobic/aerobic liquid culture of mice faeces, Gram staining and/or ADNr 16S amplification by PCR [65].

The second major limitation of gnotobiotic mouse models is that germ-free animals have a different physiology from conventional animals. For example, differences in intestinal structure (particularly intestinal peristalsis and mucus layer thickness), absorptive function, bile metabolism and behaviour have been observed. In addition, their immune system is immature, with, for example, reduced numbers of neutrophils and mucosal T cells, as well as reduced numbers of intestinal IgA-secreting plasma cells and impaired macrophage functions [64, 65]. This limits the relevance of studying immune responses in these mice, although the introduction of *C. difficile* alone or a more complex microbiota may lead to modifications of some of these factors potentially rendering the host environment more similar to that of conventional mice.

### Conventional mouse models

The main obstacle to creating an animal model of CDI is to faithfully reproduce the characteristics of human CDI, in particular, the range of disease severity seen in human cases, including symptoms, such as diarrhoea, weight loss and even fatal outcomes, although not reaching 100 % mortality rate. In this respect, mice offer a particularly suitable option. Mice are relatively resistant to *C. difficile* infection mainly due to colonization resistance provided by the resident microbiota [1]. Consequently, to induce susceptibility, several laboratories have attempted to develop mouse models of CDI based on the induction of dysbiosis with antibiotics. Three primary approaches are commonly used and will be detailed and compared in the following sections. In 2019, Shelby *et al.* proposed a standardized clinical disease score to measure disease progression in the mouse model of *C. difficile* colitis, including behavioural changes, stool characteristics and weight loss based on the symptoms observed in humans [66]. This CDI scoring system is a useful tool for standardization across studies and for enabling a more robust evaluation of vaccines and therapeutics.

### Induction of dysbiosis

#### Antibiotic cocktail model

The antibiotic cocktail model, first described by Chen *et al.* [67], involves the administration of a mixture of five antibiotics (kanamycin 0.4 mg ml^−1^, gentamicin 0.035 mg ml^−1^, metronidazole 0.215 mg ml^−1^, colistin 850 U ml^−1^ and vancomycin 0.045 mg ml^−1^) in water for 3 days. Two days after this antibiotic treatment, an intraperitoneal injection of clindamycin (10 mg kg^−1^ body weight) is given, and the mice are challenged with *C. difficile*. Depending on the *C. difficile* strains used, different levels of virulence can be observed in conventional mouse models, resulting in strain-dependent morbidity and mortality. For example, severe disease and higher mortality rates can be observed when the R20291 strain is used instead of strain 630 or VPI 10463 [68]. The severity of the disease induced also depends on the size of the inoculum used for challenge [67]. The majority of experiments deploying this model use female C57BL/6 mice. Some studies use Swiss Webster mice, such as Pensinger et *al*. in 2023, who investigated how butyrate directly affects *C. difficile* fitness [69].

#### Clindamycin model

In this model, only clindamycin is administered to induce dysbiosis by a single intraperitoneal injection of 10 mg kg^−1^ body weight, 24 h prior to infection. It is interesting to note that clindamycin alone has been shown to alter the microbiota, resulting in a high rate of colonization by *C. difficile* in one study [1], although lower colonization in another study compared to other models, and that mice deteriorate rapidly [70]. These differences could be explained by different ways in which clindamycin is administered, either orally by gavage or in the drinking water or intraperitoneally, leading to variations in the microbiota of these mice.

#### Cefoperazone model

The second model commonly used to study *C. difficile* colonization involves the use of a single broad-spectrum third-generation cephalosporin, which is cefoperazone. Typically, this antibiotic is administered in drinking water (0.5 mg ml^−1^) for 5 days (or 10 days), followed by 2 days wash out with untreated water and then challenge with *C. difficile* [71]. Compared to the antibiotic cocktail or the clindamycin model, cefoperazone induces a more prolonged dysbiosis [70]. Specifically, 6 weeks after 10 days of cefoperazone, there was a persistent change in the microbiota composition and reduced diversity. Conversely, mice exposed to the antibiotic cocktail model showed a return to eubiosis after only 4 weeks. Similar to the findings in the antibiotic cocktail model, the severity of infection varies depending on *C. difficile* strains used [72].

#### Other models

In addition to the three models described, other antibiotics have been used to induce dysbiosis and study colonization by *C. difficile* such as streptomycin or ampicillin [73,74]. While most models use young mice aged 4 to 8 weeks, some studies have used older mouse models (18 to 28 months). A recent review shows that, overall, the infection is more severe in older mice, and the innate and humoral immune responses generated are different [75].

More recently, new models have been developed, specifically to study the inflammation associated with CDI, which do not rely on the use of antibiotics. Instead, they use a genetically modified mouse, C57BL/6 IL10-/- colonized with *Helicobacter hepaticus*. The altered microbiome in this transgenic mouse makes it more susceptible to colonization by *C. difficile* [76]. This model is interesting because it resembles the case of patients with inflammatory bowel disease who are at an increased risk of CDI even in the absence of antibiotic therapy. A reporter mouse model of the NF-κB signalling pathway has also been used to test the effect of octahedral iron oxide nanocrystals in limiting spore germination and attenuating inflammation caused by *C. difficile* [77].

Through these models, it is clear that the clearance of CDI differs in different models, depending on the antibiotic used to induce the dysbiosis, and the rate of clearance is therefore associated with different impacts on the intestinal flora. Indeed, a study in 2021, comparing the effects of clindamycin, cefoperazone and streptomycin, showed that the clearance of *C. difficile* differed depending on the antibiotic because the ecological niches released for occupancy were different [73]. It should also be noted that although some studies have been carried out using the same mice lineage and the same model of dysbiosis, different microbiota have been observed, depending on the supplier/origin of the mouse colonies, leading to differences in clearance rates [78]. It is, therefore, important to take these subtleties into account when comparing the results of different studies.

### Applications

The aim of this section is to provide some recent and striking examples of the use of conventional mouse models that have contributed to new knowledge.

### Basic research into *C. difficile* physiology

The mouse model has been widely used in basic research to better understand the pathophysiology of *C. difficile* and, in particular, the molecular players involved in modulating the colonization and/or virulence of this pathogen. For example, as mentioned previously, phase variation of flagella by inversion of a DNA sequence called the flagella switch (*flg*) has recently been demonstrated to affect virulence in the conventional mouse model [27].

Bacteria adapt to osmotic stress by regulating the levels of intracellular osmolytes. Gram-positive bacteria have been shown to possess several osmolyte transport systems, many of which are under the control of the second messenger cyclic diadenosine monophosphate (c-di-AMP). Oberkampf *et al.* have shown that c-di-AMP has broad effects on *C. difficile*, including enhancing tolerance to osmotic stress, detergent-induced stress and physiologically relevant bile salts [79]. In addition, deletion of *gdpP* in *C. difficile* significantly increased intracellular concentrations of c-di-AMP. The authors used the clindamycin model and infected mice with spores of 630 WT or mutated for c-di-AMP production (Δ*gdpP*). The *C. difficile* Δ*gdpP* strain was deficient in establishing long-term colonization in mice. This deficiency was probably due to the increased susceptibility of this mutant strain to environmental stress.

### Transcriptomic and metabolomic studies

In recent years, the use of animal models has made it possible to highlight the metabolic modulations made by *C. difficile* during the colonization process in order to adapt to the ecological niche. Using a transcriptomic analysis combined with a metabolomic analysis, Jenior *et al.* [70] have shown that the ability of *C. difficile* to metabolize amino acids by Stickland fermentation could be critical and enable it to colonize an ecosystem disturbed by antibiotics. Furthermore, using the same strategy, Fletcher *et al.* [80] have shown that proline and branched-chain amino acids decrease in abundance over time consistent with their use by * C. difficile in vivo*. This opens avenues for therapies based on specific bacteria that compete for nutrients that are essential for * C. difficile* colonization.

More recently, the use of global transcriptomic approaches to monitor changes in host and *C. difficile* expression has shown that toxin-producing strains induce a unique transcriptomic signature in the context of toxin-mediated inflammation [81]. Thus, a *tcdR* mutant that does not produce toxins has a very different transcriptome. As a result, *C. difficile* metabolism is significantly altered during toxin-induced inflammation, with a remodelling of the pathways involved in carbohydrates and branched-chain amino acid metabolism.

### Microbiome and bile salt profiling

BAs have an impact on the life cycle of *C. difficile*. In particular, the presence of secondary BAs, such as chenodeoxycholic acid, is associated with resistance to *C. difficile* colonization [82]. Thus, the suppression of members of the microbiota that bio-transform primary BAs into secondary BAs by dysbiosis-inducing antibiotics favours colonization by *C. difficile*. In the cefoperazone model, the establishment of CDI was shown to correlate with changes in the microbiota and BA profile in the small and large intestines [83]. Important to note that the changes in the BA profile may vary depending on the antibiotic used to induce dysbiosis and thus modulate *C. difficile* colonization [74].

Restoring the BA profile may, therefore, appear to be a strategy to combat *C. difficile*. For example, it has been shown that the administration of *B. thetaiotaomicron* increases the concentration of DCA, which is known to inhibit *C. difficile* growth, and decreases the concentration of taurocholic acid, which promotes germination [84].

Bile salt hydrolase (BSH) enzymes are considered to be the gatekeepers of BA metabolism, reshaping the BA profile in the gut. Their main role is to deconjugate primary BAs. On this basis, a recent study [85] showed in a cefoperazone mouse model that the addition of a BSH cocktail reduced colonization. This opens up an exciting new therapeutic avenue not based on the use of antibiotics.

Another study in the cefoperazone model showed that strain R20291 induces an influx of BAs into the gut within 24 h of spore oral administration [86]. These BAs, in turn, promote the growth of *C. difficile*. Interestingly, mice receiving cholestyramine, which sequesters BAs, show a delay in colonization and a reduced level of germination of *C. difficile*. These new data suggest that *C. difficile* directly alters BA metabolism during infection.

### Diet and *C. difficile* colonization

Several studies have investigated the effect of diet on *C. difficile* colonization. It was shown in an antibiotic cocktail mouse model that the high-fat/low-fibre diet, also known as the Western diet, had a pro-*C. difficile* BA composition, i.e. more primary BAs produced to digest fats and fewer secondary BAs associated with a disruption of the microbiota [87].

A study comparing the effects of different types of fibre in the antibiotic cocktail mouse model and the VPI 10463 strain showed that pectin had a protective effect, by mainly reducing the inflammation that arises during *C. difficile* colitis and also by increasing the diversity of the microbiota. Not only fibres but protein sources also appear to have an effect. In the cefoperazone model, Yakabe *et al.* [88] showed that a soy protein-based diet increased the levels of amino acids available in the gut, thereby promoting the growth of *C. difficile*. Specifically, this diet promotes the growth of *Lactobacillus*, which can metabolize soya to release amino acids. A recent study using the clindamycin model and the Swiss Webster line [69] showed that, by using controlled diets, * C. difficile* fitness was more affected by butyrate than by acetate or propionate, demonstrating that there are also differential effects of SFCAs on *C. difficile*.

To summarize, these animal models have been instrumental in enabling the study of complex relationships between diet, host metabolism and microbiota and susceptibility to *C. difficile* infection.

### Microbiome and therapeutic molecules

The use of mouse models has allowed the therapeutic potential of new molecules to be tested and compared with existing molecules. Auranofin is an FDA-approved anti-rheumatoid arthritis drug that has been proposed as an alternative treatment because it limits *C. difficile* growth but also reduces sporulation and toxin production in the antibiotic cocktail model [89].

The use of probiotics is another interesting therapeutic approach that could be used alone or alongside antibiotics to limit microbiota perturbation. It has been shown that the cefoperazone model could be used as a platform to study these new therapeutic options [90]. The authors propose an experimental strategy that they have validated using a consortium of probiotics previously evaluated in a clinical trial in subjects treated with amoxicillin and clavulanic acid for 7 days. More recently, the antibiotic cocktail model was used to specifically test the effect of the *Bifidobacterium breve* YH68 strain in combination with vancomycin and metronidazole in a primary model of *C. difficile* infection [91]. The probiotic strain significantly reduced the additional dysbiosis caused by the anti-*C. difficile* antibiotic therapy, endowing the microbiota with earlier resilience. The clindamycin model was further used to evaluate the probiotic effect of *Bacillus velezensis* spores on CDI [92] and showed promising effects in reducing *C. difficile* 630 colonization *in vivo*. Finally, in parallel with the testing of these strains, trials using genetically modified strains are beginning. In 2022, it was shown that *Escherichia coli* Nissle 1917, long recognized as a probiotic, could be genetically modified to restore BA metabolism following post-antibiotic dysbiosis [93]. This strain was shown to inhibit spore germination and vegetative cell growth *in vitro* as well as reduce CDI *in vivo* in the antibiotic cocktail model with *C. difficile* strain VPI 10463.

In addition to testing the efficacy of probiotics in inhibiting CDI, these models have also been used to assess the effect of prebiotics [94]. For example, a team in 2021 was able to highlight the beneficial effects of β-galactosides and in particular galactosyl-β1,4-l-rhamnose on the growth of a strain of *Bifidobacterium longum* subsp. *infantis* (JCM122), which inhibits the growth of *C. difficile in vitro* and limits weight loss in a mouse model of CDI.

### Immune response and vaccination

Infection with *C. difficile* induces a strong inflammatory response, particularly through the action of toxins [95]. Major advances in our understanding of the host response mechanism are being made using knockout mouse lines for certain cytokines. A knockout mouse is a laboratory mouse in which an existing gene has been inactivated by replacing it or disrupting it with an artificial piece of DNA. For example, a recent study used the antibiotic cocktail model and the VPI 10463 strain to infect C57BL/6 WT or IL-27-deficient mice [96]. Mice lacking the IL-27 receptor showed reduced expression of cathelicidin-related antimicrobial peptide (CRAMP), which is the murine counterpart of human LL-37, after infection with *C. difficile*. Restoring CRAMP expression resulted in increased *C. difficile* clearance and decreased mortality in IL-27 receptor-deficient mice following *C. difficile* challenge.

Conventional mouse models were also used to determine the contribution of the immune adaptive response to *C. difficile* clearance. Interestingly, in cefoperazone-treated mice deficient in B and T lymphocytes (RAG1-/-), it was still possible to observe clearance of *C. difficile* [97]. This appears to be dependent on the dysbiosis model, as a previous publication using a neomycin, metronidazole and vancomycin cocktail for 72 h showed that RAG1-/- mice failed to clear the infection following faecal transplantation [98].

Conventional mouse models have also contributed to the development of vaccine strategies, although these are often tested in the hamster model. For example, purified CD0873, a *C. difficile* lipoprotein, was shown to protect against long-term persistence in a conventional murine active immunization model, with a corresponding specific adaptive immune response to CD0873 [43]. More recently, using the antibiotic cocktail model and spores of strain R20291, it has been shown that colonization can also be prevented by immunization with a preparation of FliCD, a recombinant fusion protein between the FliC and FliD proteins of *C. difficile* [99].

In conclusion, conventional mouse models of *C. difficile* infection vary widely. As a result, it can sometimes be difficult to compare studies that do not follow exactly the same protocol whether it is the choice of the mouse strain and the dysbiosis to which it is subjected or the choice of the *C. difficile* strain and its mode of administration (spores or vegetative cells), as well as the amount of inoculum used.

## Relapse models

While the first description of an *in vivo* model of *C. difficile* infection in mice was published in 2008 [67], recurrence models were not available until 2011 [100]. Similar to the terminology used to describe CDI in humans, *in vivo* models of recurrence can be divided into two distinct groups: reinfection models and relapse models. In both cases, mice will develop two successive CDI episodes, but the mechanisms involved in the development of the second CDI episode will vary.

In relapse models, the second CDI episode is induced by exposing mice to relapse-inducing triggers such as antibiotics. Depending on the timing of this exposure, relapse models can be further separated into two categories, which we will describe as ‘early relapse models’ and ‘late relapse models’. In early relapse models, mice are exposed to the relapse-inducing trigger during the course of the initial CDI episode. In most cases, this trigger is applied 3 or 4 days after *C. difficile* administration. In comparison, for late relapse models, the relapse-inducing trigger is applied after the first episode has naturally resolved.

In reinfection models, the second CDI episode is obtained after a second administration of *C. difficile* by oral gavage. While reinfection models have been mentioned in a few studies [67,100], they will not be further addressed in this review due to their limited use. Furthermore, to our knowledge, recurrence models have not been used in gnotobiotic mice. As such, this section will focus on relapse models, both early and late, in conventional mice.

### Early relapse models

To our knowledge, the first publication reporting an early relapse model was in 1987 for hamster models [101] and 2015 for mice models [102]. In the work from 2015, authors used a 5-day vancomycin treatment starting 4 days after the initial *C. difficile* 630 challenge, which is the most frequently used model for early relapses. Using vancomycin as a relapse-inducing trigger is appropriate considering that (i) vancomycin is amongst the first lines of treatment for human CDI [103] and (ii) vancomycin treatment leads to relapses in 25 % of patients [104]. In this model, vancomycin treatment resolved the initial CDI episode between day 4 and day 9 (decreased *C. difficile* shedding, toxin production and histological lesions in the caecum and colon) [102]. However, within 2 days of vancomycin treatment completion, signs of CDI were reported (increased *C. difficile* shedding, toxin levels in faeces and histopathological damage scores for the caecum and colon), suggesting relapse of infection [102]. Interestingly, in this work [102], *C. difficile* carriage in the faeces remained slightly above the detection limits after vancomycin treatment completion, suggesting incomplete clearance. Of note, the initial CDI episode in this work was done following the cefoperazone model [72], which is known to lead to prolonged dysbiosis and *C. difficile* carriage [70]. Similarly, continued *C. difficile* shedding after vancomycin completion has been reported in other studies [68]. For example, one study used three different models for the initial episode and compared the impact of relapse induced by vancomycin on these models, using three *C. difficile* strains (630, R20291 and VPI 10463). In all three models, relapse occurred in a significant proportion of treated mice. Specifically, 80–100 % of mice relapsed across strains and models, with the exception of VPI 10463 in the Chen model (57 % relapses as opposed to 100% and 83 % with strains R20291 and 630, respectively) [68]. The model used to induce the initial CDI did not appear to influence toxin production or spore shedding during relapse [68]. The main difference reported across the models was related to clinical symptoms. Higher rates of diarrhoea were reported during relapse when the initial CDI was induced by cefoperazone rather than with the antibiotic cocktail. In addition, the development of diarrhoea occurred homogenously across mice when the initial CDI was induced by cefoperazone (day 10 to day 11), while its development spread over several days when the initial CDI was induced using the antibiotic cocktail (day 9 to day 12) [68].

Early relapse models have been used in several studies investigating the relapse rates of new therapeutic strategies for CDI treatment. Indeed, the first publication using a mouse early relapse model investigated whether faecal microbiota transplantation (FMT) could prevent relapse [102]. Since then, FMT has been included in the therapeutic arsenal for CDI, particularly for patients suffering from multiple recurrent CDI episodes [103]. Similarly, such models have been used to investigate whether probiotic treatments can limit the rate of recurrence. For instance, hamsters were used in an early relapse model to show that administration of *Saccharomyces boulardii* leads to reduced *C. difficile* burden, decreased toxin litres in faeces and increased hamster survival after relapse induction through vancomycin treatment [101]. In another study, mice were used in an early relapse model to study the impact of a combination of *Lactobacillus acidophilus* NCFM, *Lactobacillus paracasei* Lpc-37, *Bifidobacterium lactis* Bi-07 and *Bifidobacterium lactis* B1-04 [90]. Finally, early relapse models in hamsters and mice have also been used to investigate novel therapeutics, such as amixicile [105], auranofin [21,106], HSGN-218 [107] and most recently CDBN-YGXZ [21,29, 101].

### Late relapse models

In late relapse models, mice are usually exposed to the relapse-inducing trigger more than 20 days after the initial *C. difficile* challenge, at which point *C. difficile* is often either below or near the threshold level for detection [67]. Common relapse-inducing triggers used for late relapse models are clindamycin [56,100] and vancomycin [108,109].

The first late relapse model was published in 2011 by Sun and colleagues, using the *C. difficile* UK1 strain [100]. In this publication, the initial CDI episode was induced using the antibiotic cocktail described by Chen *et al.* [67], and mice that survived 30 days after the initial *C. difficile* challenge were then exposed to the relapse-inducing trigger: three clindamycin intraperitoneal injections at day 30, 31 and 32 [100]. In this study, clindamycin injections were capable of inducing relapses in all mice within 24 h after the first injection. Clinical manifestations included diarrhoea, which resolved within 2 days, and increased toxin production and spore shedding in the faeces, with the highest intensity reached 3 days after the first injection. Interestingly, the relapses did not appear to be associated with significant weight loss, as opposed to the initial CDI episode [100].

While early relapse models have been used to investigate new therapies, late relapse models have mostly been used to study the physiology of relapses, either from the bacterial or host side. For instance, late relapse models have been used to study the effect of para-cresol production by *C. difficile* on the host microbiota and CDI relapses [109]. Para-cresol is a bacteriostatic phenolic compound produced by tyrosine fermentation, which can be produced by *C. difficile* using enzymes encoded on the *hpdBCA* operon [109]. The authors used a para-cresol-deficient 630Δ*erm* mutant and compared the occurrence of relapse for mice after vancomycin exposure from day 21 to day 28. In their model, mice from both groups showed low levels of *C. difficile* carriage at day 21 after a cefoperazone-induced initial CDI, and *C. difficile* carriage became below the limit of detection for mice of both groups after 7 days of vancomycin treatment. Upon completion of the vancomycin treatment, relapses were detected through increased spore shedding and reached 100 % relapse rate for both groups 4 days after completion of the vancomycin course. However, clinical signs of disease were not reported. Furthermore, authors reported a lower burden for mice that had received the para-cresol-deficient *C. difficile* strain, suggesting that para-cresol production by *C. difficile* is involved in *in vivo* fitness during relapses [109].

Late relapse models have also been used to assess the contribution of sporulation in CDI relapses. In a study published by Deakin and colleagues, it was shown that mice infected with the sporulation-deficient *spo0A* mutant of *C. difficile* did not develop CDI relapses after vancomycin exposure (oral administration from day 20 to day 27), while relapses could be detected for mice infected with either the R20291 or 630Δ*erm* parental strains [108]. Interestingly, authors could not culture *C. difficile* from the intestinal tracts of mice during vancomycin treatment, while *C. difficile* could be cultured from cages, bedding and chews. The authors concluded that sporulation contributes to relapses by allowing the persistence of *C. difficile* in the environment, although they could not fully rule out the presence of non-culturable forms of *C. difficile* from the intestinal tract [108].

Finally, as noted for early relapse models, late relapse models have also been applied in hamsters [6]. It was shown that relapses could be induced by a single clindamycin intraperitoneal injection 3 weeks after the initial episode. While hamsters infected with the R20291 parental strain became moribund and had to be humanely euthanized within 2 days, none of the hamsters infected with a mutant strain deficient in cellobiose utilization showed evidence of relapses and *C. difficile* could not be cultivated from caecal contents harvested from sacrificed hamsters. In this work, authors linked the decrease in relapse to the poor sporulation capacity of the cellobiose utilization-deficient mutant, which is consistent with the work by Deakin *et al.* [108].

### Advantages and limitations

**Fig. 1.**
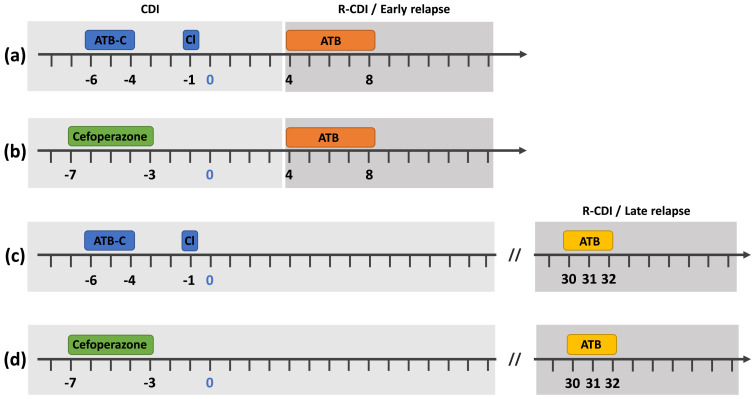
Schematic representation of the main *in vivo* relapse models. Simplified representation of the four *in vivo* relapse models based on the model used for the primary CDI episode as well as the timing of relapse studied: (a) initial CDI induced with the antibiotic cocktail followed by early relapse induction, (b) initial CDI induced with cefoperazone followed by early relapse induction, (c) initial CDI induced with the antibiotic cocktail followed by late relapse induction and (d) initial CDI induced with cefoperazone followed by late relapse induction. Administration of *C. difficile* by oral gavage is represented as day 0. ATB-C = antibiotic cocktail (see Chen *et al.* [67]), Cl = clindamycin injection, ATB = single antibiotic treatment.

To conclude, as discussed in this section, extensive *in vivo* models for the study of CDI relapses are relatively recent, and there are multiple models available (see Table 1 for a summary of the main studies published in each model and Fig. 1 for a schematic representation of the models). As such, there are many parameters to examine in order to select the models and parameters that fit best the scientific question being addressed. First, authors need to consider which animal model is most appropriate. While most studies have applied relapse models to conventional mice, several studies have applied relapse models to hamsters, for early and late relapse models. The limited use of relapse models in hamsters can be explained by the very high rate of hamster mortality during the initial CDI episode (80 –100 %). The consequences of this are either (i) a limitation in the number of animals remaining to investigate relapse, therefore decreasing the statistical power of the study, or (ii) the need for larger animal cohort sizes, which raises ethical concerns. As such, it appears that most studies can be done using the mice model of relapses, unless specific concerns justify using hamster-based models of relapse. Second, authors then need to select the relapse model, including the timing of relapse induction and the type of relapse-inducing trigger. As indicated before, early relapse models are mainly used for investigating relapse rates in response to new therapies, while late relapse models are more suited to studying bacterial and/or host factors involved in the physiology of relapses. However, when designing early relapse assays, researchers should be particularly careful in selecting the duration of the relapse-inducing trigger. Indeed, relapses are not limited to treatment failures. As such, relapse-inducing triggers applied during the acute phase of infection should be tailored to be as close as possible to efficient treatment guidelines. Otherwise, the model should be considered as a treatment-failure model rather than an early relapse model. Finally, as deployed for characterizing initial CDIs, there are multiple parameters to track for relapses including clinical signs of infection and disease severity (weight loss, diarrhoea, poor fur coat and mortality) as well as bacteriological signs of infection [bacterial shedding (spores or vegetative cells), toxin production, histopathological damage and microbiota composition]. To fully understand the relapses occurring in the model selected, studies should be designed to include monitoring with standardized clinical scoring sheets as for primary CDI and bacteriological status.

**Table 1.** Studies using the main relapse models

Primary CDI	Secondary CDI	
	Early relapse	Late relapse
Antibiotic cocktail model [67]	Warren *et al.* 2012 [105]Abutaleb *et al.* 2020 [106]Naclerio *et al.* 2022 [107]Hu *et al.* 2023 [29]	Sun *et al.* 2011 [100]Collins *et al.* 2015 [56]
Cefoperazone model [72]	Seekatz *et al.* 2015 [102]De Wolfe *et al.* 2019 [90]Abutaleb and Seleem 2021 [21]*	Passmore *et al*. 2018 [109]
Clindamycin model	Elmer and McFarland 1987 [101]*	Deakin *et al.* 2012 [108]Hasan *et al.* 2021 [6]*

Publications are organized according to the model used for primary CDI and the timing of relapse studied (early or late), with asterisks (*) indicating studies using hamsters.

One of the main limitations that should be considered when designing relapse studies is to ensure that the infection generated is indeed a relapse of the initial infection rather than a reinfection from spores released into the animal cages after the primary infection. As such, researchers specifically interested in understanding relapses should include specific measures to minimize the presence of spores in the animal environment when the relapse-inducing trigger is applied, such as increased frequency of cage changes. Finally, for late relapse models, bacterial clearance should be carefully monitored between the first infectious episode and the relapse to determine whether a genuine bacteriological clearance is obtained or not. As such, the choice of the primary CDI model is paramount. Indeed, as described earlier, the rate of *C. difficile* clearance during the first episode varies between the cefoperazone and cocktail models [70]. Thus, researchers should be particularly careful when selecting models for late relapse, ensuring *C. difficile* clearance can be confirmed before applying the relapse-inducing trigger.

## Conclusion

Understanding the pathogenesis of *C. difficile* has progressed steadily since the development of the hamster and mouse models of CDI. Although the hamster model was one of the first models used in *C. difficile* research and remains a useful and important model for the study of *C. difficile*, advances in other animal models offer new opportunities. In recent years, we have also seen the emergence of studies using non-mammalian models, such as *C. elegans*, the fruit fly, the worm or the zebrafish reviewed in Hu and Garey [110].

**Fig. 2.**
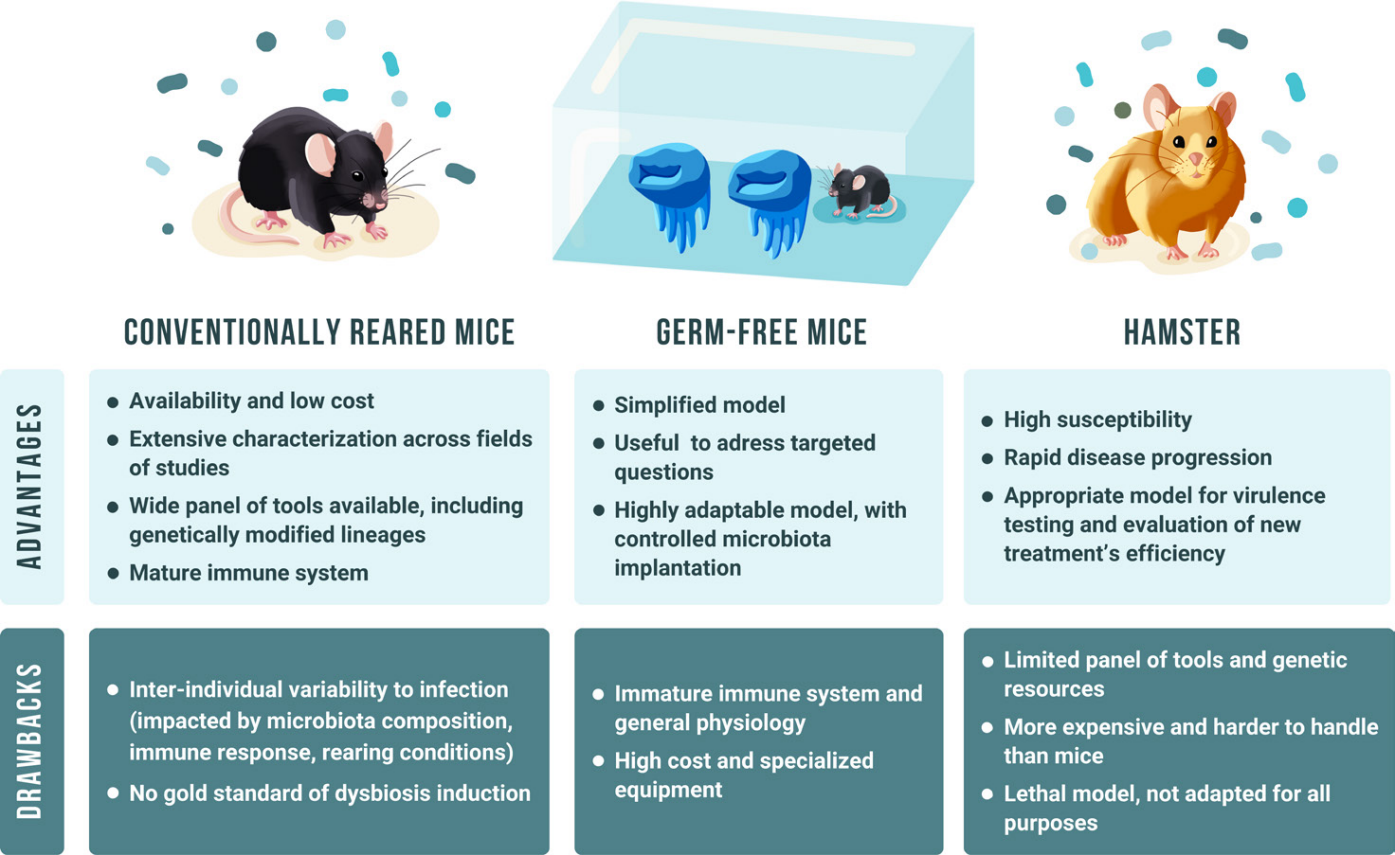
Comparison of advantages and drawbacks of different rodent models of CDI.

It is clear that the choice of model can have a significant impact on the results obtained in *C. difficile* research, but standardization of methods remains a key issue and is essential to generate clear and reproducible data. Rodent animal models of CDI, although complementary, have advantages and disadvantages, as shown in Fig. 2, and it is likely that the use of different animal models will be re-evaluated in the coming years, particularly as more is learnt about the complex relationship between *C. difficile*, the host, the gut microbiota and the immune response to CDI.

Due to significant differences in susceptibility to CDI in different animal models, caution should be exercised when comparing results obtained from different models. Importantly, the relevance of data obtained from any model to human CDI remains paramount.

## Abbreviations

BA: bile acid
BAs: bile acids
BSH: bile salt hydrolase
CDCA: chenodeoxycholic acid
CDI: *C. difficile* infection
c-di-AMP: cyclic diadenosine monophosphate
CFU: Colony Forming Unit
CRAMP: cathelicidin-related antimicrobial peptide
DCA: deoxycholic acid
FMT: faecal microbiota transplantation
GT: glucosyltransferase
HEPA: High-Efficiency Particulate Air
KO: knockout
MIC: minimum inhibitory concentration
TcdA: toxin A
TcdB: toxin B

## References

[1] Lawley TD, Clare S, Walker AW, Goulding D, Stabler RA (2009). Antibiotic treatment of clostridium difficile carrier mice triggers a supershedder state, spore-mediated transmission, and severe disease in immunocompromised hosts. Infect Immun.

[2] Price AB, Larson HE, Crow J (1979). Morphology of experimental antibiotic-associated enterocolitis in the hamster: a model for human pseudomembranous colitis and antibiotic-associated diarrhoea. Gut.

[3] Douce G, Goulding D, Mullany P, Roberts AP Clostridium difficile.

[4] Best EL, Freeman J, Wilcox MH (2012). Models for the study of *Clostridium difficile* infection. Gut Microbes.

[5] Sambol SP, Tang JK, Merrigan MM, Johnson S, Gerding DN (2001). Infection of hamsters with epidemiologically important strains of *Clostridium difficile*. J Infect Dis.

[6] Hasan MK, Dhungel BA, Govind R (2021). Characterization of an operon required for growth on cellobiose in *Clostridioides difficile*. Microbiology.

[7] Small JD (1968). Fatal enterocolitis in hamsters given lincomycin hydrochloride. Lab Anim Care.

[8] Larson HE, Borriello SP (1990). Quantitative study of antibiotic-induced susceptibility to *Clostridium difficile* enterocecitis in hamsters. Antimicrob Agents Chemother.

[9] Merrigan M, Sambol S, Johnson S, Gerding DN (2003). Susceptibility of hamsters to human pathogenic *Clostridium difficile* strain B1 following clindamycin, ampicillin or ceftriaxone administration. Anaerobe.

[10] Weiss W, Pulse M, Vickers R (2014). In vivo assessment of SMT19969 in a hamster model of *Clostridium difficile* infection. Antimicrob Agents Chemother.

[11] Chiari EF, Weiss W, Simon MR, Kiessig ST, Pulse M (2021). Oral immunotherapy with human secretory immunoglobulin A improves survival in the hamster model of *Clostridioides difficile* infection. J Infect Dis.

[12] Burdet C, Sayah-Jeanne S, Nguyen TT, Miossec C, Saint-Lu N (2017). Protection of hamsters from mortality by reducing fecal moxifloxacin concentration with DAV131A in a model of moxifloxacin-induced *Clostridium difficile* colitis. Antimicrob Agents Chemother.

[13] Ghose C, Eugenis I, Edwards AN, Sun X, McBride SM (2016). Immunogenicity and protective efficacy of *Clostridium difficile* spore proteins. Anaerobe.

[14] Rätsep M, Kõljalg S, Sepp E, Smidt I, Truusalu K (2017). A combination of the probiotic and prebiotic product can prevent the germination of *Clostridium difficile* spores and infection. Anaerobe.

[15] Diniz AN, Moura LNF, Cruz DSG, Oliveira Junior CA, Figueiredo HCP (2022). Characterization of the virulence of three novel clade 2 *Clostridioides* (*Clostridium*) *difficile* strains and a two-year screening in animals and humans in Brazil. PLoS One.

[16] Wang Y, Wang S, Bouillaut L, Li C, Duan Z (2018). Oral immunization with nontoxigenic *Clostridium difficile* strains expressing chimeric fragments of TcdA and TcdB elicits protective immunity against *C. difficile* infection in both mice and hamsters. Infect Immun.

[17] Nale JY, Spencer J, Hargreaves KR, Buckley AM, Trzepiński P (2016). Bacteriophage combinations significantly reduce *Clostridium difficile* growth in vitro and proliferation in vivo. Antimicrob Agents Chemother.

[18] Zhang B-Z, Cai J, Yu B, Hua Y, Lau CC (2016). A DNA vaccine targeting TcdA and TcdB induces protective immunity against *Clostridium difficile*. BMC Infect Dis.

[19] Miezeiewski M, Schnaufer T, Muravsky M, Wang S, Caro-Aguilar I (2015). An in vitro culture model to study the dynamics of colonic microbiota in Syrian golden hamsters and their susceptibility to infection with *Clostridium difficile*. ISME J.

[20] Wilson KH, Sheagren JN, Freter R (1985). Population dynamics of ingested *Clostridium difficile* in the gastrointestinal tract of the Syrian hamster. J Infect Dis.

[21] Abutaleb NS, Seleem MN (2021). In vivo efficacy of auranofin in a hamster model of *Clostridioides difficile* infection. Sci Rep.

[22] Koon HW, Su B, Xu C, Mussatto CC, Tran DH-N (2016). Probiotic *Saccharomyces boulardii* CNCM I-745 prevents outbreak-associated *Clostridium difficile*-associated cecal inflammation in hamsters. Am J Physiol Gastrointest Liver Physiol.

[23] Bilverstone TW, Garland M, Cave RJ, Kelly ML, Tholen M (2020). The glucosyltransferase activity of *C. difficile* Toxin B is required for disease pathogenesis. PLoS Pathog.

[24] Karyal C, Hughes J, Kelly ML, Luckett JC, Kaye PV (2021). Colonisation factor CD0873, an attractive oral vaccine candidate against *Clostridioides difficile*. Microorganisms.

[25] Borriello SP, Ketley JM, Mitchell TJ, Barclay FE, Welch AR (1987). *Clostridium difficile*--a spectrum of virulence and analysis of putative virulence determinants in the hamster model of antibiotic-associated colitis. J Med Microbiol.

[26] Hong HA, Ferreira WT, Hosseini S, Anwar S, Hitri K (2017). The spore coat protein CotE facilitates host colonization by *Clostridium difficile*. J Infect Dis.

[27] Trzilova D, Warren MAH, Gadda NC, Williams CL, Tamayo R (2022). Flagellum and toxin phase variation impacts intestinal colonization and disease development in a mouse model of *Clostridioides difficile* infection. Gut Microbes.

[28] Simpson M, Bilverstone T, Leslie J, Donlan A, Uddin MJ (2023). *Clostridioides difficile* binary toxin binding component increases virulence in a hamster model. Open Forum Infect Dis.

[29] Hu X, Dong R, Huang S, Zeng Y, Zhan W (2023). CDBN-YGXZ, a novel small-molecule drug, shows efficacy against *Clostridioides difficile* infection and recurrence in mouse and hamster infection models. Antimicrob Agents Chemother.

[30] Phan JR, Do DM, Truong MC, Ngo C, Phan JH (2022). An aniline-substituted bile salt analog protects both mice and hamsters from multiple *Clostridioides difficile* strains. Antimicrob Agents Chemother.

[31] Bhute SS, Mefferd CC, Phan JR, Ahmed M, Fox-King AE (2022). A high-carbohydrate diet prolongs dysbiosis and *Clostridioides difficile* carriage and increases delayed mortality in a hamster model of infection. Microbiol Spectr.

[32] Onderdonk AB, Cisneros RL, Bartlett JG (1980). *Clostridium difficile* in gnotobiotic mice. Infect Immun.

[33] Vernet A, Corthier G, Dubos-Ramaré F, Parodi AL (1989). Relationship between levels of *Clostridium difficile* toxin A and toxin B and cecal lesions in gnotobiotic mice. Infect Immun.

[34] Corthier G, Muller MC, Wilkins TD, Lyerly D, L’Haridon R (1991). Protection against experimental pseudomembranous colitis in gnotobiotic mice by use of monoclonal antibodies against *Clostridium difficile* toxin A. Infect Immun.

[35] Depitre C, Delmee M, Avesani V, L’Haridon R, Roels A (1993). Serogroup F strains of *Clostridium difficile* produce toxin B but not toxin A. J Med Microbiol.

[36] Corthier G, Dubos F, Raibaud P (1985). Modulation of cytotoxin production by *Clostridium difficile* in the intestinal tracts of gnotobiotic mice inoculated with various human intestinal bacteria. Appl Environ Microbiol.

[37] Reeves AE, Koenigsknecht MJ, Bergin IL, Young VB (2012). Suppression of *Clostridium difficile* in the gastrointestinal tracts of germfree mice inoculated with a murine isolate from the family Lachnospiraceae. Infect Immun.

[38] Girinathan BP, DiBenedetto N, Worley JN, Peltier J, Arrieta-Ortiz ML (2021). Invivo commensal control of *Clostridioides difficile* virulence. Cell Host Microbe.

[39] Ng KM, Ferreyra JA, Higginbottom SK, Lynch JB, Kashyap PC (2013). Microbiota-liberated host sugars facilitate post-antibiotic expansion of enteric pathogens. Nature.

[40] Ferreyra JA, Wu KJ, Hryckowian AJ, Bouley DM, Weimer BC (2014). Gut microbiota-produced succinate promotes *C. difficile* infection after antibiotic treatment or motility disturbance. Cell Host Microbe.

[41] Spigaglia P, Barketi-Klai A, Collignon A, Mastrantonio P, Barbanti F (2013). Surface-layer (S-layer) of human and animal *Clostridium difficile* strains and their behaviour in adherence to epithelial cells and intestinal colonization. J Med Microbiol.

[42] Kink JA, Williams JA (1998). Antibodies to recombinant *Clostridium difficile* toxins A and B are an effective treatment and prevent relapse of C. difficile-associated disease in A hamster model of infection. Infect Immun.

[43] Bradshaw WJ, Bruxelle J-F, Kovacs-Simon A, Harmer NJ, Janoir C (2019). Molecular features of lipoprotein CD0873: a potential vaccine against the human pathogen *Clostridioides difficile*. J Biol Chem.

[44] Giordano N, Hastie JL, Smith AD, Foss ED, Gutierrez-Munoz DF (2018). Cysteine desulfurase IscS2 plays a role in oxygen resistance in *Clostridium difficile*. Infect Immun.

[45] Janoir C, Denève C, Bouttier S, Barbut F, Hoys S (2013). Adaptive strategies and pathogenesis of *Clostridium difficile* from in vivo transcriptomics. Infect Immun.

[46] Kansau I, Barketi-Klai A, Monot M, Hoys S, Dupuy B (2016). Deciphering adaptation strategies of the epidemic *Clostridium difficile* 027 strain during infection through in vivo transcriptional analysis. PLoS One.

[47] Pruss KM, Sonnenburg JL (2021). *C. difficile* exploits a host metabolite produced during toxin-mediated disease. Nature.

[48] Pruss KM, Enam F, Battaglioli E, DeFeo M, Diaz OR (2022). Oxidative ornithine metabolism supports non-inflammatory *C. difficile* colonization. Nat Metab.

[49] Castex F, Jouvert S, Bastide M, Corthier G (1994). Kinetics of appearance of intestinal lesions in mice mono-associated with a lethal or non-lethal strain of *Clostridium difficile*. J Med Microbiol.

[50] Soavelomandroso AP, Gaudin F, Hoys S, Nicolas V, Vedantam G (2017). Biofilm structures in a mono-associated mouse model of *Clostridium difficile* infection. Front Microbiol.

[51] Studer N, Desharnais L, Beutler M, Brugiroux S, Terrazos MA (2016). Functional intestinal bile acid 7α-dehydroxylation by *Clostridium scindens* associated with protection from *Clostridium difficile* infection in a gnotobiotic mouse model. Front Cell Infect Microbiol.

[52] Péchiné S, Janoir C, Boureau H, Gleizes A, Tsapis N (2007). Diminished intestinal colonization by *Clostridium difficile* and immune response in mice after mucosal immunization with surface proteins of *Clostridium difficile*. Vaccine.

[53] Battaglioli EJ, Hale VL, Chen J, Jeraldo P, Ruiz-Mojica C (2018). *Clostridioides difficile* uses amino acids associated with gut microbial dysbiosis in a subset of patients with diarrhea. Sci Transl Med.

[54] Pu M, Cho JM, Cunningham SA, Behera GK, Becker S (2021). Plasmid acquisition alters vancomycin susceptibility in *Clostridioides difficile*. Gastroenterology.

[55] Kumar R, Maynard CL, Eipers P, Goldsmith KT, Ptacek T (2016). Colonization potential to reconstitute a microbe community in patients detected early after fecal microbe transplant for recurrent *C. difficile*. BMC Microbiol.

[56] Collins J, Auchtung JM, Schaefer L, Eaton KA, Britton RA (2015). Humanized microbiota mice as a model of recurrent *Clostridium difficile* disease. Microbiome.

[57] Hugenholtz F, de Vos WM (2018). Mouse models for human intestinal microbiota research: a critical evaluation. Cell Mol Life Sci.

[58] Dabard J, Dubos F, Martinet L, Ducluzeau R (1979). Experimental reproduction of neonatal diarrhea in young gnotobiotic hares simultaneously associated with *Clostridium difficile* and other Clostridium strains. Infect Immun.

[59] Czuprynski CJ, Johnson WJ, Balish E, Wilkins T (1983). Pseudomembranous colitis in *Clostridium difficile*-monoassociated rats. Infect Immun.

[60] Schmidt DJ, Beamer G, Tremblay JM, Steele JA, Kim HB (2016). A tetraspecific VHH-based neutralizing antibody modifies disease outcome in three animal models of *Clostridium difficile* infection. Clin Vaccine Immunol.

[61] Thran M, Pönisch M, Danz H, Horscroft N, Ichtchenko K (2023). Co-administration of an effector antibody enhances the half-life and therapeutic potential of RNA-encoded nanobodies. Sci Rep.

[62] Herzog MK-M, Cazzaniga M, Peters A, Shayya N, Beldi L (2023). Mouse models for bacterial enteropathogen infections: insights into the role of colonization resistance. Gut Microbes.

[63] Pavao A, Graham M, Trofimuk O, Delaney ML, Yeliseyev V (2022). Protocol for using negative pressure isolator systems to study BSL-2 organisms in gnotobiotic murine models. STAR Protoc.

[64] Smith K, McCoy KD, Macpherson AJ (2007). Use of axenic animals in studying the adaptation of mammals to their commensal intestinal microbiota. Semin Immunol.

[65] Bhattarai Y, Kashyap PC (2016). Germ-free mice model for studying host-microbial interactions. Methods Mol Biol.

[66] Shelby RD, Tengberg N, Conces M, Olson JK, Navarro JB (2020). Development of a standardized scoring system to assess a murine model of *Clostridium difficile* colitis. J Invest Surg.

[67] Chen X, Katchar K, Goldsmith JD, Nanthakumar N, Cheknis A (2008). A mouse model of *Clostridium difficile*-associated disease. Gastroenterology.

[68] Castro-Córdova P, Díaz-Yáñez F, Muñoz-Miralles J, Gil F, Paredes-Sabja D (2020). Effect of antibiotic to induce *Clostridioides difficile*-susceptibility and infectious strain in a mouse model of *Clostridioides difficile* infection and recurrence. Anaerobe.

[69] Pensinger DA, Fisher AT, Dobrila HA, Van Treuren W, Gardner JO (2023). Butyrate differentiates permissiveness to *Clostridioides difficile* infection and influences growth of diverse *C. difficile* isolates. Infect Immun.

[70] Jenior ML, Leslie JL, Young VB, Schloss PD (2018). *Clostridium difficile* alters the structure and metabolism of distinct cecal microbiomes during initial infection to promote sustained colonization. mSphere.

[71] Reeves AE, Theriot CM, Bergin IL, Huffnagle GB, Schloss PD (2011). The interplay between microbiome dynamics and pathogen dynamics in a murine model of *Clostridium difficile* infection. Gut Microbes.

[72] Theriot CM, Koumpouras CC, Carlson PE, Bergin II, Aronoff DM (2011). Cefoperazone-treated mice as an experimental platform to assess differential virulence of *Clostridium difficile* strains. Gut Microbes.

[73] Schubert AM, Sinani H, Schloss PD (2021). Clearance of *Clostridioides difficile* colonization is associated with antibiotic-specific bacterial changes. mSphere.

[74] Buffie CG, Bucci V, Stein RR, McKenney PT, Ling L (2015). Precision microbiome reconstitution restores bile acid mediated resistance to *Clostridium difficile*. Nature.

[75] Shin JH, Pawlowski SW, Warren CA (2021). Teaching old mice new tricks: the utility of aged mouse models of *C. difficile* infection to study pathogenesis and rejuvenate immune response. Gut Microbes.

[76] Abernathy-Close L, Barron MR, George JM, Dieterle MG, Vendrov KC (2021). Intestinal inflammation and altered gut microbiota associated with inflammatory bowel disease render mice susceptible to *Clostridioides difficile* colonization and infection. mBio.

[77] Lee W-T, Wu Y-N, Chen Y-H, Wu S-R, Shih T-M (2017). Octahedron iron oxide nanocrystals prohibited *Clostridium difficile* spore germination and attenuated local and systemic inflammation. Sci Rep.

[78] Tomkovich S, Stough JMA, Bishop L, Schloss PD (2020). The initial gut microbiota and response to antibiotic perturbation influence *Clostridioides difficile* clearance in mice. mSphere.

[79] Oberkampf M, Hamiot A, Altamirano-Silva P, Bellés-Sancho P, Tremblay YDN (2022). c-di-AMP signaling is required for bile salt resistance, osmotolerance, and long-term host colonization by *Clostridioides difficile*. Sci Signal.

[80] Fletcher JR, Erwin S, Lanzas C, Theriot CM (2018). Shifts in the gut metabolome and *Clostridium difficile* transcriptome throughout colonization and infection in a mouse model. mSphere.

[81] Fletcher JR, Pike CM, Parsons RJ, Rivera AJ, Foley MH (2021). *Clostridioides difficile* exploits toxin-mediated inflammation to alter the host nutritional landscape and exclude competitors from the gut microbiota. Nat Commun.

[82] Theriot CM, Koenigsknecht MJ, Carlson PE, Hatton GE, Nelson AM (2014). Antibiotic-induced shifts in the mouse gut microbiome and metabolome increase susceptibility to *Clostridium difficile* infection. Nat Commun.

[83] Koenigsknecht MJ, Theriot CM, Bergin IL, Schumacher CA, Schloss PD (2015). Dynamics and establishment of *Clostridium difficile* infection in the murine gastrointestinal tract. Infect Immun.

[84] Li X, Kang Y, Huang Y, Xiao Y, Song L (2021). A strain of Bacteroides thetaiotaomicron attenuates colonization of *Clostridioides difficile* and affects intestinal microbiota and bile acids profile in a mouse model. Biomed Pharmacother.

[85] Foley MH, Walker ME, Stewart AK, O’Flaherty S, Gentry EC (2023). Bile salt hydrolases shape the bile acid landscape and restrict *Clostridioides difficile* growth in the murine gut. Nat Microbiol.

[86] Wexler AG, Guiberson ER, Beavers WN, Shupe JA, Washington MK (2021). *Clostridioides difficile* infection induces a rapid influx of bile acids into the gut during colonization of the host. Cell Rep.

[87] Hazleton KZ, Martin CG, Orlicky DJ, Arnolds KL, Nusbacher NM (2022). Dietary fat promotes antibiotic-induced *Clostridioides difficile* mortality in mice. NPJ Biofilms Microbiom.

[88] Yakabe K, Higashi S, Akiyama M, Mori H, Murakami T (2022). Dietary-protein sources modulate host susceptibility to *Clostridioides difficile* infection through the gut microbiota. Cell Rep.

[89] Hutton ML, Pehlivanoglu H, Vidor CJ, James ML, Thomson MJ (2019). Repurposing auranofin as a *Clostridioides difficile* therapeutic. J Antimicrob Chemother.

[90] De Wolfe TJ, Kates AE, Barko L, Darien BJ, Safdar N (2019). Modified mouse model of *Clostridioides difficile* infection as a platform for probiotic efficacy studies. Antimicrob Agents Chemother.

[91] Yang J, Meng L, Yang H (2022). Therapeutic effects of bifidobacterium breve YH68 in combination with vancomycin and metronidazole in a primary *Clostridioides difficile*-infected mouse model. Microbiol Spectr.

[92] Larsen IS, Chenaux M, Collins FWJ, Mandic A, Hansen LBS (2023). Bacillus velezensis DSM 33864 reduces *Clostridioides difficile* colonization without disturbing commensal gut microbiota composition. Sci Rep.

[93] Koh E, Hwang IY, Lee HL, De Sotto R, Lee JWJ (2022). Engineering probiotics to inhibit *Clostridioides difficile* infection by dynamic regulation of intestinal metabolism. Nat Commun.

[94] Hirano R, Sakanaka M, Yoshimi K, Sugimoto N, Eguchi S (2021). Next-generation prebiotic promotes selective growth of bifidobacteria, suppressing *Clostridioides difficile*. Gut Microbes.

[95] Madan R, Petri WA (2012). Immune responses to *Clostridium difficile* infection. Trends Mol Med.

[96] Xu B, Wu X, Gong Y, Cao J (2021). IL-27 induces LL-37/CRAMP expression from intestinal epithelial cells: implications for immunotherapy of *Clostridioides difficile* infection. Gut Microbes.

[97] Leslie JL, Vendrov KC, Jenior ML, Young VB (2019). The gut microbiota is associated with clearance of *Clostridium difficile* infection independent of adaptive immunity. mSphere.

[98] Littmann ER, Lee J-J, Denny JE, Alam Z, Maslanka JR (2021). Host immunity modulates the efficacy of microbiota transplantation for treatment of *Clostridioides difficile* infection. Nat Commun.

[99] Wang S, Ju X, Heuler J, Zhang K, Duan Z (2023). Recombinant fusion protein vaccine containing *Clostridioides difficile* FliC and FliD protects mice against *C. difficile* infection. Infect Immun.

[100] Sun X, Wang H, Zhang Y, Chen K, Davis B (2011). Mouse relapse model of *Clostridium difficile* infection. Infect Immun.

[101] Elmer GW, McFarland LV (1987). Suppression by *Saccharomyces boulardii* of toxigenic *Clostridium difficile* overgrowth after vancomycin treatment in hamsters. Antimicrob Agents Chemother.

[102] Seekatz AM, Theriot CM, Molloy CT, Wozniak KL, Bergin IL (2015). Fecal microbiota transplantation eliminates *Clostridium difficile* in a murine model of relapsing disease. Infect Immun.

[103] van Prehn J, Reigadas E, Vogelzang EH, Bouza E, Hristea A (2021). European Society of Clinical Microbiology and Infectious Diseases: 2021 update on the treatment guidance document for *Clostridioides difficile* infection in adults. Clin Microbiol Infect.

[104] Louie TJ, Miller MA, Mullane KM, Weiss K, Lentnek A (2011). Fidaxomicin versus vancomycin for *Clostridium difficile* infection. N Engl J Med.

[105] Warren CA, van Opstal E, Ballard TE, Kennedy A, Wang X (2012). Amixicile, a novel inhibitor of pyruvate: ferredoxin oxidoreductase, shows efficacy against *Clostridium difficile* in a mouse infection model. Antimicrob Agents Chemother.

[106] Abutaleb NS, Seleem MN (2020). Auranofin, at clinically achievable dose, protects mice and prevents recurrence from *Clostridioides difficile* infection. Sci Rep.

[107] Naclerio GA, Abutaleb NS, Li D, Seleem MN, Sintim HO (2020). Ultrapotent inhibitor of *Clostridioides difficile* growth, which suppresses recurrence in vivo. J Med Chem.

[108] Deakin LJ, Clare S, Fagan RP, Dawson LF, Pickard DJ (2012). The *Clostridium difficile* spo0A gene is a persistence and transmission factor. Infect Immun.

[109] Passmore IJ, Letertre MPM, Preston MD, Bianconi I, Harrison MA (2018). Para-cresol production by *Clostridium difficile* affects microbial diversity and membrane integrity of Gram-negative bacteria. PLoS Pathog.

[110] Hu C, Garey KW (2023). Nonmammalian models to study *Clostridioides difficile* infection; a systematic review. Anaerobe.

